# Impact of Snow on Vegetation Green-Up on the Mongolian Plateau

**DOI:** 10.3390/plants14152310

**Published:** 2025-07-26

**Authors:** Xiang Zhang, Chula Sa, Fanhao Meng, Min Luo, Xulei Wang, Xin Tian, Endon Garmaev

**Affiliations:** 1 College of Geographic Science, Inner Mongolia Normal University, Hohhot 010022, China; 20226016006@mails.imnu.edu.cn (X.Z.); mfh320@imnu.edu.cn (F.M.); luomin@imnu.edu.cn (M.L.); 20224016063@mails.imnu.edu.cn (X.W.); 20224016029@mails.imnu.edu.cn (X.T.); 2 Key Laboratory of Mongolian Plateau Geographical Research, Inner Mongolia Autonomous Region, Hohhot 010022, China; 3 Baikal Institute of Nature Management, Siberian Branch of the Russian Academy of Sciences, 670047 Ulan-Ude, Russia; garend1@yandex.ru

**Keywords:** snow, vegetation index, SOS, impact mechanism, Mongolia Plateau

## Abstract

Snow serves as a crucial water source for vegetation growth on the Mongolian Plateau, and its temporal and spatial variations exert profound influences on terrestrial vegetation phenology. In recent years, global climate change has led to significant changes in snow and vegetation start of growing season (SOS). Therefore, it is necessary to study the mechanism of snow cover on vegetation growth and changes on the Mongolian Plateau. The study found that the spatial snow cover fraction (SCF) of the Mongolian Plateau ranged from 50% to 60%, and the snow melt date (SMD) ranged from day of the year (DOY) 88 to 220, mainly concentrated on the northwest Mongolian Plateau mountainous areas. Using different SOS methods to calculate the vegetation SOS distribution map. Vegetation SOS occurs earlier in the eastern part compared to the western part of the Mongolian Plateau. In this study, we assessed spatiotemporal distribution characteristics of snow on the Mongolian Plateau over the period from 2001 to 2023. The results showed that the SOS of the Mongolian Plateau was mainly concentrated on DOY 71-186. The Cox survival analysis model system established SCF and SMD on vegetation SOS. The SCF standard coefficient is 0.06, and the SMD standard coefficient is 0.02. The SOS_NDVI_ coefficient is −0.15, and the SOS_NDGI_ coefficient is −0.096. The results showed that the vegetation SOS process exhibited differential response characteristics to snow driving factors. These research results also highlight the important role of snow in vegetation phenology and emphasize the importance of incorporating the unique effects of vegetation SOS on the Mongolian Plateau.

## 1. Introduction

Global warming is exerting a certain effect on ecosystems, with snow cover patterns poised to have impacts across vegetation [1,2,3]. In the past decade, changes in snow cover have significantly decreased, and climate change has had a negative impact on snow cover changes around the world. In winter, snow covers nearly half of the northern hemisphere’s land surface and plays an important role in hydrological and biogeochemical processes [4,5,6]. Global climate change has led to earlier vegetation phenology in spring [7,8]. Research has shown that the temporal changes in vegetation phenology during the spring period profoundly affect ecosystem functions, including carbon fixation capacity [9], drought stress recovery [10], and surface evapotranspiration processes. Through regulating the carbon water flux and energy exchange mechanisms between land and air systems, it forms a feedback regulatory effect on the climate system. The biosphere and the atmosphere highlight the pivotal role of phenological changes in global climate change research. As one of the most sensitive climate indicators, snow cover has undergone significant changes in vegetation ecosystems over the past few decades.

The spatiotemporal changes in snow cover can seriously affect vegetation dynamics, thereby altering vegetation SOS. Specifically, snow cover affects vegetation SOS through various physiological and vegetation green-up processes [11]. Snow can improve soil moisture and promote vegetation growth, SOS. In addition, delayed snow melting usually leads to a delay in the spring vegetation green-up period and a shortened growth season, thereby limiting vegetation SOS. Understanding the interaction between snow cover and vegetation SOS is crucial for gaining a deeper understanding of climate feedback mechanisms, which has recently attracted widespread attention.

The vegetation pattern of the Mongolian Plateau is deeply influenced by the continental arid climate and complex terrain, forming a rich and diverse grassland ecosystem. According to the water, dominant plant composition, and community structure, the main vegetation can be mainly divided into three vegetation types [12]. Meadow steppe, distributed in the eastern and northern edge areas with relatively abundant precipitation, with high vegetation coverage and dense grass groups, mainly consisting of mesophilic or drought-tolerant steppe and miscellaneous. Typical steppe is the most widely distributed on the Mongolian Plateau, occupying a vast area in the central and eastern regions. The dominant species are typical drought-tolerant clustered steppe, with a relatively uniform community structure and moderate productivity. Desert steppe, distributed in the western and southern Mongolian Plateau regions with scarce precipitation, is a transitional zone from grassland to desert. The vegetation is sparse and low, mainly consisting of drought-tolerant steppe and small semi-shrubs, showing strong adaptability to drought stress.

The Mongolian Plateau is located in the arid and semi-arid regions of East Asia, and its unique geographical unit is sensitive to climate change [5]. Its temperate continental climate forms a unique vegetation type. In addition, the grassland ecosystem of the Mongolian Plateau plays a crucial role in the carbon cycle in East Asia [13]. Multispectral time-series analysis enabled by satellite remote sensing has revolutionized the monitoring of snow-vegetation SOS interactions, particularly in quantifying SOS responses to cryosphere dynamics across the Mongolian Plateau [14,15,16,17]. In addition, early spring snowmelt has a good effect on different vegetation types growth on the Mongolian Plateau.

The study of climate factors that affect vegetation phenology considers the effects of snow on the Mongolian Plateau. On the basis of analyzing the spatiotemporal evolution of snow and the vegetation green-up period, this paper will further quantify the impact mechanism of snow change on the vegetation green-up period in different vegetation types and have certain theoretical and perspective innovations in deepening the understanding of the snow-vegetation relationship. In this study, we used remote sensing data to achieve snow coverage and combined it with vegetation indices to achieve high-precision calculation of the vegetation green-up period on the Mongolian Plateau. We evaluated the fit of the survival model and linear regression model, analyzed the impact of snow parameters on vegetation phenology and their interaction factors, and summarized the threshold effect of snow on vegetation phenology using survival analysis methods.

## 2. Materials and Methods

### 2.1. Study Area

**Figure 1:**
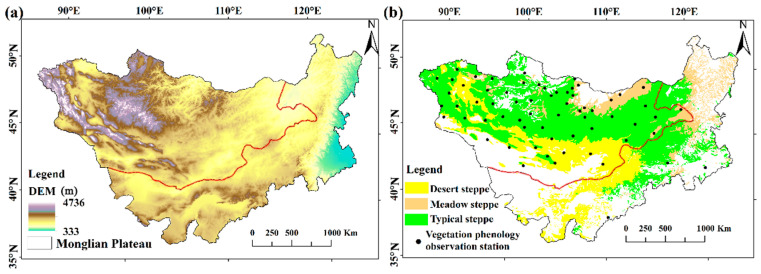
Study area: (**a**) DEM of the Mongolian Plateau; (**b**) Distribution of different steppe types.

The Mongolian Plateau includes all of Mongolia and Inner Mongolia, China, with coordinates 37°46′–53°08′ N and 87°40′–122°15′ E [18]. This area features an arid and semi-arid climate. As a unique geographical unit, the research area is characterized by mountainous geomorphological features [19]. The Mongolian Plateau features diverse vegetation types, which are primarily categorized into meadow steppe, typical steppe, and desert steppe, as indicated in Figure 1 and Table 1.

### 2.2. Data Sources

Utilizing the International Geosphere-Biosphere Programme (IGBP) classification system, this study employed yearly 2020 MODIS MCD12Q1 land cover data (500 m spatial resolution) to delineate temperate ecoregions across the Mongolian Plateau. The identified land cover data demonstrate distribution of vegetation types (https://lpdaacsvc.cr.usgs.gov/appeears/task/area, accessed on 27 June 2022).

Vegetation indices time-series reconstruction incorporated Savitzky-Golay filtering from January 2001 to December 2023 8-day (DOY 1 and DOY 361 with 8-day interval) MOD09A1 with 500 m spatial resolution detection of phonological transition dates (https://lpdaacsvc.cr.usgs.gov/appeears/task/area, accessed on 23 November 2024).

MOD10A1-derived January 2001 to December 2023 SCF with 1-day data (DOY 1- DOY 365) with 500 m spatial resolution were studied. (https://lpdaacsvc.cr.usgs.gov/appeears/task/area, accessed on 27 June 2022). Snow melt date (SMD) refers to the last DOY of 5 continuous days with the SCF in a hydrological year.

### 2.3. Methods

#### 2.3.1. Snow Accumulation Variation Parameters

SMD refers to the occurrence of SCF for five consecutive days within a hydrological year with a period from September 1st of the current year to August 31st of the following year [20]. The normalized difference snow index (NDSI), serving as an indicator for the monitoring of snow, has an impact on the growth of vegetation and the productivity of the ecosystem during the season. The monthly SCF was determined by means of the maximum value synthesis approach. The average value method was used to calculate the multi-year winter values. According to the formula provided in the MODIS snow product MOD10A1 user guide, we determined the SCF for the study area [21]. The formula is as follows:

(1) $$SCF=\left(-0.01+\left(1.45\times NDSI\right)\right)\times 100\%$$

#### 2.3.2. Vegetation Index

The normalized difference vegetation index (NDVI) is calculated as the normalized form of the red ($\rho_{red}$) and NIR ($\rho_{NIR}$) band reflectance. This vegetation index is sensitive to soil background; its normalized ratio can effectively suppress radiation differences caused by instrument calibration, solar angle, terrain, cloud shadow, and atmospheric condition changes, thereby enhancing the vegetation signal [22]:

(2) $$NDVI=\frac{\rho_{NIR}-\rho_{red}}{\rho_{NIR}+\rho_{red}}$$

The Enhanced Vegetation Index (EVI) is an indicator based on remote sensing technology for monitoring and evaluating vegetation conditions, widely used in the fields of ecology, agriculture, forestry, and environmental science. EVI has enhanced its sensitivity to vegetation cover through optimization algorithms, especially in high-coverage areas and arid and semi-arid regions. The EVI adds a blue band ($\rho_{Blue}$) to enhance vegetation signals and correct the effects of soil background and aerosol scattering [23].

(3) $$EVI=2.5\times \frac{\rho_{NIR}-\rho_{red}}{\rho_{NIR}+6\times \rho_{red}-7.5\times \rho_{Blue}+1}$$

Normalized phenological vegetation index (NDPI) band combination leverages differential absorption characteristics: red band, near infrared band, and short wavelength infrared band, achieving accuracy in separating snow/soil mixtures under sparse vegetation. The advantages of NDPI mainly come from its ability to overcome the adverse effects of soil background heterogeneity, explaining that NDPI has a high correlation with leaf moisture content due to the addition of shortwave infrared bands, while the correlation with other vegetation indices is relatively small.

(4) $$NDPI=\frac{\rho_{NIR}-\left[\alpha \times \rho_{red}+\left(1-\alpha \right)\times \rho_{SWIR}\right]}{\rho_{NIR}+\left[\alpha \times \rho_{red}+\left(1-\alpha \right)\times \rho_{SWIR}\right]}$$

The weight coefficient α, which ranges between 0.0 and 1.0, is determined based on the specific spectral configuration of the satellite sensor. The MODIS spectral bandwidth parameter is 0.74 [24,25].

The normalized difference greenness index (NDGI) is a remote sensing index used to quantify the greenness of surface vegetation based on the ratio of the difference in reflectance between near-infrared and green light bands to their sum. The range of its values is −1 to 1, with higher values indicating denser surface vegetation coverage and stronger greenness. NDGI has application value in vegetation dynamic monitoring, land use/cover change research, and environmental monitoring and assessment. The green, red, and near-infrared spectral bands are comprehensively utilized by the semi-analytical NDGI index to capture the unique spectral features of vegetation [26]. The NDGI calculation is as follows:

(5) $$NDGI=\frac{\alpha \times \rho_{Green}+\left(1-\alpha \right)\times \rho_{NIR}-\rho_{red}}{\alpha \times \rho_{Green}+\left(1-\alpha \right)\times \rho_{NIR}+\rho_{red}}$$

where weighting coefficient α is sensor-specific, with MODIS datasets requiring empirical derivation of this parameter to account for platform-dependent spectral response characteristics, α is 0.65 in reference.

#### 2.3.3. Calculation of Vegetation SOS

The annual metrics of SOS_NDVI_, SOS_EVI_, SOS_NDPI_, and SOS_NDGI_ were computed through per-pixel temporal to quantify phenological progression dynamics. Then, the cumulative SOS_NDVI_, SOS_EVI_, SOS_NDPI_, and SOS_NDGI_. The dataset four-parameter logistic curve modeling (Formulas (6) and (7)) within a scientifically robust framework [27], with parametric optimization achieved through curvature extrema analysis. The vegetation SOS was subsequently determined as the temporal coordinate corresponding to the absolute curvature minimum in the fitted function.

(6) $$y\left(t\right)=\frac{c}{1+e^{a+bt}}+d$$

(7) $$z=e^{a+bt}$$

Here t is DOY, y(t) is the time t cumulative vegetation index value; the fitting parameters include a and b; c is the difference between the maximum cumulative NDVI and the background NDVI; d is the background NDVI value; and z is the parameter [28].

The β_max_ method operationalizes the quantification of temporal growth dynamics for NDVI, EVI, NDPI, and NDGI indices through first-derivative analysis (β) derived from logistic curvature modeling. This method defines vegetation SOS temporal position as attaining maximum first-derivative magnitude (β_max_) within the vegetation [29].

To analyze the dynamic changes in regional vegetation, we conducted processing work based on the 16-day time series vegetation index data obtained. Firstly, we calculated the average vegetation index values for each spatial pixel in the time series for each 16-day period. On this basis, we further conducted a time series analysis on these average vegetation index value sequences arranged in chronological order, calculated their rate of change to quantify the rate and direction of vegetation state evolution over time at each pixel location, and then calculated the following:

(8) $${VI}_{ratio}\left(t\right)=\frac{{VI}_{t+1}-{VI}_{t}}{{VI}_{t}}$$

where ${VI}_{t}$ is the average vegetation index value at a given time t calculated over many years. The maximum ${VI}_{ratio}\left(t\right)$ value from this multi-year averaged vegetation index series is selected as a common threshold. This threshold is applied uniformly to annual NDVI, EVI, NDPI, and NDGI data for the same pixel.

To overcome the limitations of traditional phenological parameters in characterizing the nonlinear characteristics of vegetation growth, this study introduces the curvature change rate (CCR) as the core analysis indicator for the first time. The CCR_max_ method curvature change rate (CCR) is calculated for fitting the logistic function as follows [30]:

(9) $$CCR=b^{3}cz\left\{\frac{3z\left(1-z\right){\left(1+z\right)}^{3}\left[2{\left(1+z\right)}^{2}+b^{2}c^{2}z\right]}{{\left[{\left(1-z\right)}^{4}+{\left(bcz\right)}^{2}\right]}^{2.5}}-\frac{{\left(1+z\right)}^{2}\left(1+2z-{5z}^{2}\right)}{{\left[{\left(1+z\right)}^{4}+{\left(bcz\right)}^{2}\right]}^{1.5}}\right\}$$

In this context, the parameters of a, b, and c are identical to what they are in Equation (6). The time that corresponds to the initial local maximum value of the CCR serves as the indicator for the start of the vegetation SOS.

Based on the G20 method, the SOS is defined as the day when the vegetation ratio first reaches the dynamic threshold of 0.2 in the fitted logistic function [31]. Regardless of the specific vegetation type growing in a particular location, a vegetation ratio of 0.2 means that the location has reached 20% of its maximum green-up level, and this rule applies to the calculation of annual vegetation ratio.

According to the methodology developed by the G20, vegetation SOS refers to the date on which the vegetation ratio first reaches the dynamic threshold of 0.2 during the fitting of the logistic function [32]. From the perspective of vegetation, a vegetation ratio of 0.2 represents that the vegetation at a specific location has achieved 20%, and this determination is not related to the actual vegetation type present at that location. The calculation of the annual vegetation ratio is carried out as follows:

(10) $${VI}_{ratio}=\frac{{VI}_{t}-{VI}_{min}}{{VI}_{max}-{VI}_{min}}=0.2$$

here, the values at time t refer to the NDVI, EVI, NDPI, and NDGI, respectively. Meanwhile, VI_max_ and VI_min_ represent the maximum and minimum values of the NDVI, EVI, NDPI, and NDGI, respectively, within the range of annual time series data in the rising phase.

#### 2.3.4. Accuracy Assessment

The accuracy of vegetation greening periods obtained by different vegetation indices and vegetation SOS methods was evaluated by comparing ground observation vegetation SOS data with the Chinese Ecosystem Research Network (CREN) (http://www.cnern.org.cn/, accessed on 25 November 2024) and the Institute of Geography and Ecological Geology of the Mongolian Academy of Sciences. Although there are differences in vegetation SOS observed by satellites and ground observations, satellite remote sensing and ground observation trends have been validated to possess consistency over time and in different spatial locations. Calculating the average value using satellite remote sensing vegetation SOS value centered on each ground-measured station, which forms a pair with the vegetation SOS period of the ground observation station. Then, a simple linear regression model was used to analyze all vegetation SOS period data from 2001 to 2023 in order to obtain four indicators for each vegetation index: regression slope, root mean square error (RMSE), and bias are used as key evaluation indicators. The differences between vegetation SOS extracted by different methods were explained. We carried out additional calculations and made comparisons of the threshold percentages related to the amplitudes of the vegetation index curves. These threshold percentages correspond to both the SOS of vegetation as observed on the ground and the SOS of vegetation as detected by satellites.

#### 2.3.5. Survival Analysis Model

Survival analysis models were first formulated within the medical domain, mainly with the aim of evaluating the time span from the beginning to the end of an individual’s life [32]. This model provides a quantitative analysis framework for intervention effectiveness by tracking the time course of events and has become an important statistical tool. Survival analysis models have unique advantages in the field of data statistics, with their core ability being to accurately handle complex data involving event occurrence times. They are particularly adept at addressing issues. In vegetation phenology research, this model can be innovatively used to quantitatively analyze the temporal dynamics of the key ecological event of vegetation green-up [33].

The key structure of the survival analysis model revolves around the risk function h(t). This function assesses the instantaneous probability density of the subject under study when it is in the critical state of an event occurring at the specific time point t. In this practical research concerning vegetation phenology, we incorporated the Cox proportional hazards model [34]. The Cox model is actually a simple model, and its goal is to evaluate the impact of each variable on survival time, specifically referring to the time at which any event that requires consideration of time occurs. In survival analysis research, for certain instances that occur during our study period without any time, we refer to this situation as censoring. The main premise of the Cox model is to assume that the risk ratio is a fixed value; that is, the impact of covariates on survival probability does not change over time. The purpose of the Cox model is to simultaneously evaluate the impact of several factors on survival. In other words, it allows us to examine how specific factors affect the incidence of specific events at specific time points. We quantitatively analyzed the dynamic correlation between environmental covariates and phenology events. This model does not require the specific parameter form of the risk function h_0_(t). This method effectively addresses the situation of data overlap at observation time nodes by accumulating the weighted average of risk exposure at event time points and effectively separates time effects and covariate effects through the partial likelihood estimation method. It is particularly suitable for evaluating the nonlinear driving mechanism of dynamic environmental factors such as temperature and humidity on the timing of vegetable SOS.

(11) $$h\left(t\right)=h_{0s}e^{\sum_{i=1}^{p}\left|\beta_{i}X_{i}+\delta_{i}X_{i}ln\left(t\right)\right|}$$

where s represents stratification; t represents the DOY; h_0_(t) represents the baseline hazard; X represents the influencing variables; $\beta_{i}$ and $\delta_{i}$ are the coefficients that correspond to each influencing variable and its time-dependent component, respectively.

This study adopts a two-stage parameter estimation strategy to construct a survival analysis model. Firstly, based on the principle of maximum likelihood estimation, the model parameters ($\beta_{i}$ and $\delta_{i}$) are asymptotically and effectively estimated. This process optimizes the partial likelihood function to achieve statistical inference of covariate effects; the Breslow non-parametric estimator Breslow (1974) [35] was used to reconstruct the benchmark risk function h_0_(t). The model parameter $\beta_{i}$ characterizes the linear effect of covariates while $\delta_{i}$ captures the dynamic changes of covariate effects on a logarithmic scale over time. Together, they form the risk ratio function $e^{\beta_{i}+\delta_{i}ln\left(t\right)}$. This parameterization method is particularly suitable for analyzing the nonlinear time-driving effects of meteorological factors on the rate of vegetation SOS.

This study adopts a methodological framework that combines standardized preprocessing with the Cox proportional hazards model. At the same time, based on the reconstruction of the original data, the original data are established to provide a quantitative basis for constructing an operable green-up date prediction equation. In the model, we integrated 2001–2023 green-up dates retrieved from SOS_NDVI_, SOS_EVI_, SOS_NDPI_, and SOS_NDGI_.

## 3. Results

### 3.1. Variation of Snow Parameters

**Figure 2:**
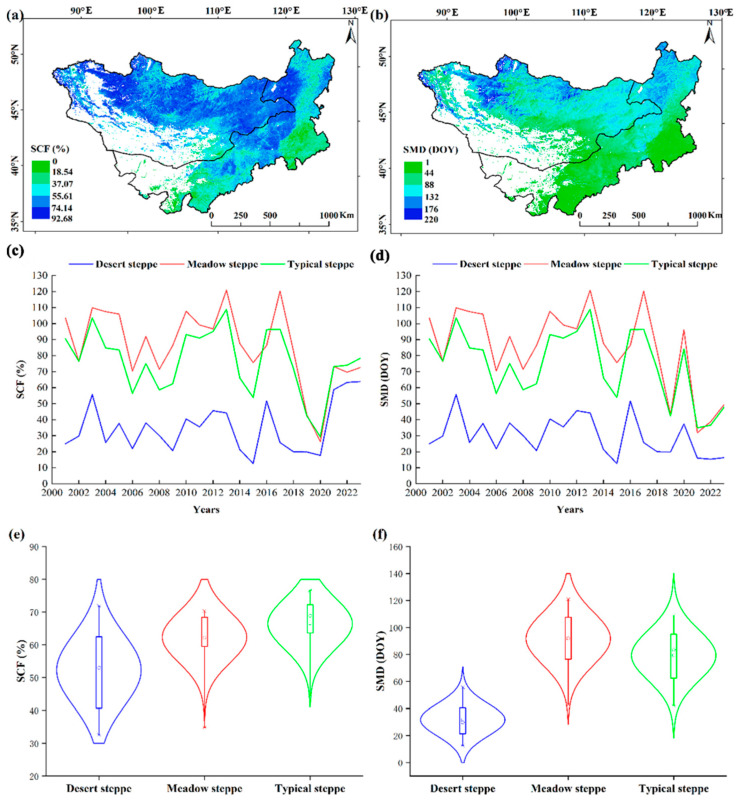
The SCF and SMD from 2001 to 2023 for different vegetation types across the Mongolian Plateau: (**a**,**b**) The spatial map that shows the distribution of the average SCF and SMD; (**c**,**d**) the trend of change for the SCF and SMD; (**e**,**f**) the violin plot presenting the spatial statistics of the SCF and SMD.

The SCF dynamics across the Mongolian Plateau over the 2001–2023 period reveal distinct spatiotemporal patterns (Figure 2a). Statistical analyses indicate a decline in SCF across the entire Mongolian Plateau. Geospatial stratification demonstrates SCF 50–60% in the northwestern Mongolian Plateau’s mountainous region, contrasting sharply with the southeastern semi-arid region. The spatial SCF distribution corresponds to zonal vegetation characteristics, with 78.3% of observed patterns with typical meadow and temperate steppe parameters. Specifically, the meadow steppe region, mainly located in the north, has an average SCF of 62.0%. The typical steppe area exhibits an average SCF of 65.99%. In the typical steppe region, the average SCF falls within the range of 29.36% to 76.62%. Furthermore, Figure 2b clearly illustrates the SMD changes of different vegetation types. It is clearly observable that the SCF deficit experiences a consistent decline across the entire study area. From Figure 2b, it can be seen that it depicts the spatial distribution pattern of SMD on the Mongolian Plateau, which gradually decreases from north to south. Areas with SMD exceeding DOY 105 are concentrated in the north, indicating prolonged snow cover and a relatively later spring snowmelt in this region. Figure 2c shows the temporal variations of SCF in different vegetation types. The SCF of meadow steppe and typical steppe on the Mongolian Plateau shows a slow downward trend. Figure 2d shows the temporal variations of SMD in different vegetation types. The SMD of meadow steppe and typical steppe on the Mongolian Plateau show from DOY 30 to 120. The violin plot in Figure 2e displays the statistics of the SCF. Specifically, the desert steppe has an average SCF of 47.26%, with the highest value reaching 89%. On the other hand, meadow steppe exhibits an average SCF of 66.26%, and the highest value that was observed reaches 91.9%. As shown in Figure 2f, the average SMD corresponding to desert steppe is DOY 25. In addition, the average value of SMD is at the level of DOY 94 in meadow steppe, and its highest value is DOY 204.

### 3.2. Comparison of Spatial Different Vegetation Green-Up Methods

**Figure 3:**
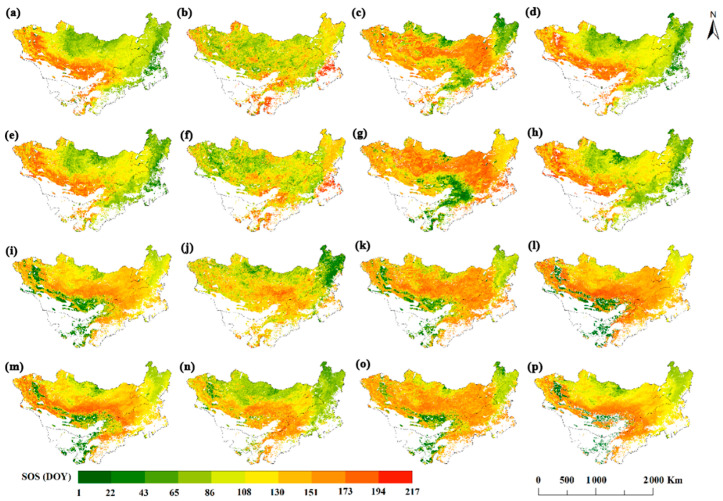
A 2001–2023 vegetation SOS over the Mongolia Plateau: (**a**–**d**) SOS_NDVI_ β_max,_ RC_max_, CCR_max_, and G20 method; (**e**–**h**) SOS_EVI_ β_max_, RC_max_, CCR_max,_ and G20 method; (**i**–**l**) SOS_NDPI_ β_max,_ RC_max_, CCR_max,_ and G20 method; (**m**–**p**) SOS_NDGI_ β_max,_ RC_max_, CCR_max,_ and G20 method.

We conducted an analysis of the SOS distribution of different vegetation types using four vegetation indices and four green-up extraction methods across the Mongolian Plateau over the period from 2001 to 2023 (Figure 3a–p). Based on the normalized difference vegetation index, four different green-up extraction methods are presented. Located in the northern and eastern directions of the Mongolian Plateau, the average SOS_NDVI_ was found to be within the range of DOY 72 to 180 in Figure 3a–d. The SOS_NDVI_ values for desert vegetation are predominantly located in the western regions of the Mongolian Plateau. Here, Figure 3e–h shows the average SOS_EVI_ above DOY 71-186, with the northern part of the Mongolian Plateau as its main distribution area. Figure 3i–l show the spatial distribution of the mean SOS_NDPI_ with four green-up extraction methods. Here, the average SOS_NDPI_, which is above DOY 97-182 with a main distribution range, covers the northern region of the Mongolian Plateau. Figure 3m–p depicts the spatial spread of the average SOS based on the SOS_NDGI_, which is derived from four different green-up extraction methods.

**Figure 4:**
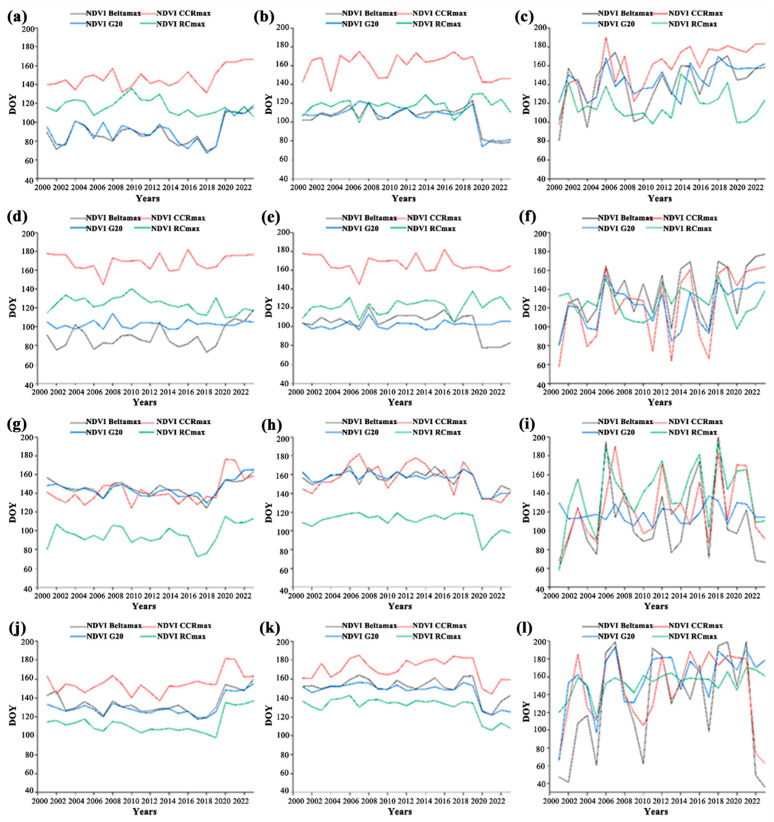
A 2001 to 2023 different vegetation types SOS across the Mongolia Plateau: (**a**–**c**) The changing trend of SOS_NDVI_ for the meadow, typical, and desert vegetation types; (**d**–**f**) SOS_EVI_ change trend meadow, typical, and desert vegetation types; (**g**–**i**) SOS_NDPI_ change trend meadow, typical, and desert vegetation types; (**j**–**l**) SOS_NDGI_ change trend meadow, typical, and desert vegetation types.

As depicted in Figure 4a–l, it is shown that various vegetation types exhibit identical characteristics regarding the SOS, with a consistent annual decreasing trend. Specifically, within the meadow steppe, the average SOS based on the SOS_NDVI_ DOY falls within the range of 67 to 167. In the case of the typical steppe, the average SOS_NDVI_ DOY spans from 74 to 175. Moreover, in the desert steppe area, as illustrated in Figure 4a–c, the average SOS_NDVI_ is found to be between DOY 80 and 191. According to the information in Figure 4d–f, the average start of the season based on the meadow steppe is within the range of DOY 75 to 182. In the typical steppe, the average SOS_EVI_ DOY is from 77 to 182. As shown in Figure 4d–f, the average SOS_EVI_ DOY ranges from 57 to 177 in the desert steppe area. In Figure 4g–i, the average SOS_NDPI_ DOY ranges from 72 to 176 in the meadow steppe. In a typical steppe environment, the average value range of SOS_NDPI_ DOY covers from 79 to 178. In the desert steppe area, as shown in Figure 4g–i, the average SOS_NDPI_ DOY falls within the range of 58 to 204. In Figure 4j–l, it is indicated that the average SOS_NDGI_ DOY in the meadow steppe is from 98 to 164. For typical steppe, the average SOS_NDGI_ DOY fluctuates between 77 and 182. Moreover, for the desert steppe area, according to Figure 4g–i, the average SOS_NDGI_ DOY is between 36 and 199.

**Figure 5:**
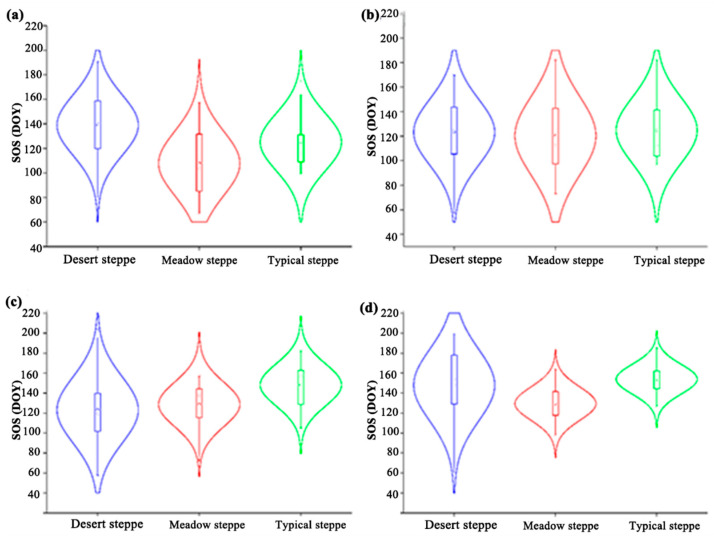
A 2001–2023 vegetation SOS over the Mongolia Plateau: (**a**–**d**) SOS_NDVI_, SOS_EVI_, SOS_NDPI_, and SOS_NDGI_ violin plots.

In different vegetation types, the vegetation SOS extracted by the four vegetation indices was statistically analyzed, as shown in Figure 5a–d. In Figure 5a, the average value of using NDVI combined with four calculations of vegetation methods is obtained as SOS_NDVI_. In desert steppe, the average value of SOS_NDVI_ DOY is 140. In meadow steppe, the average value of SOS_NDVI_ DOY is 108. In typical steppe, the average value of SOS_NDVI_ DOY is 125. In Figure 5b, the average value of using EVI combined with four calculations of vegetation methods is obtained as SOS_EVI_. In desert steppe, the average value of SOS_EVI_ DOY is 123. In meadow steppe, the average value of SOS_EVI_ DOY is 120. In typical steppe, the average value of SOS_EVI_ DOY is 125. In Figure 5c, the average value of using NDPI combined with four calculations of vegetation methods is obtained as SOS_NDPI_. In desert steppe, the average value of SOS_NDPI_ DOY is 123. In meadow steppe, the average value of SOS_NDPI_ DOY is 129. In typical steppe, the average value of SOS_NDPI_ DOY is 125. In Figure 5d, the average value of using NDGI combined with four calculations of vegetation methods is obtained as SOS_NDGI_. In desert steppe, the average value of SOS_NDGI_ DOY is 148. In meadow steppe, the average value of SOS_NDGI_ DOY is 129. In typical steppe, the average value of SOS_NDGI_ DOY is 154.

**Figure 6:**
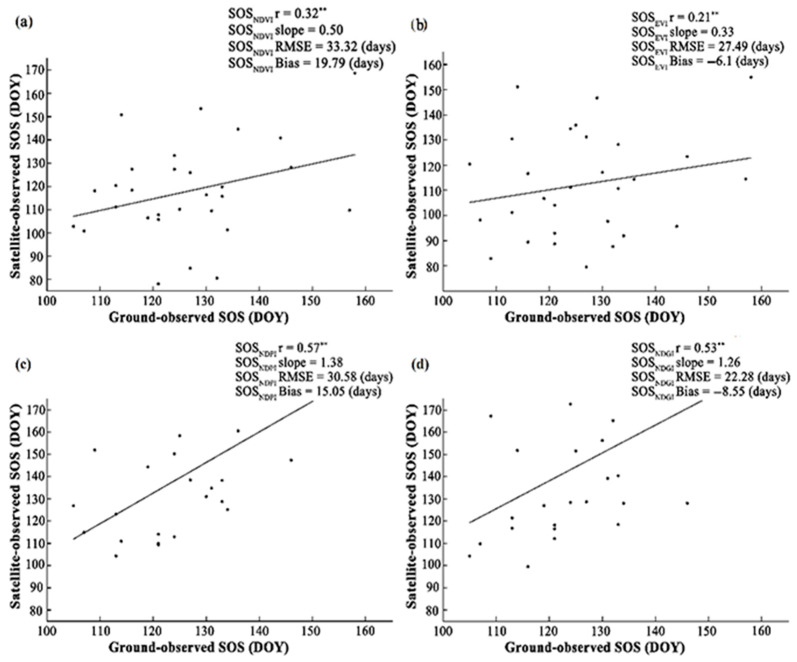
Compare the performance of vegetation growth SOS obtained from four vegetation indices using four extracted from four vegetation indices for all station years. (**a**–**d**) For 2001–2023, ground observation SOS and satellite SOS_NDVI_, SOS_EVI_, SOS_NDPI,_ and SOS_NDGI_ scatter plots for all station years. SOS_NDVI_ sample size: 28, SOS_EVI_ sample size: 29, SOS_NDPI_ sample size: 20, and SOS_NDGI_ sample size: 22. The symbols ** represent significance levels at 0.1 and 0.01, respectively.

We focused on studying and comparing the SOS_NDVI_, SOS_EVI_, SOS_NDPI_, and SOS_NDGI_. The comparison between ground observation vegetation phenology station and satellite observation vegetation SOS validated by four methods shows a significant correlation for all years (Figure 6a–d). The SOS_NDVI_ is relatively low, with the highest RMSE (r = 0.32 **, RMSE = 33.32 days). We make a comparison of the interannual fluctuations and tendencies of vegetation SOS under different vegetation indices (Figure 6a). SOS_EVI_ obtained the most accurate vegetation extraction of the SOS method; the characteristic is that r has reached its maximum value, while RMSE is in a state of minimum value (r = 0.21 **, RMSE = 27.49 days) (Figure 6b). The SOS_NDPI_ observed on the ground observation vegetation phenology is 0.57 ** (*p* < 0.01), with a detected vegetation SOS of 1.38 days/year (*p* < 0.01) (Figure 6c). The SOS_NDGI_ observed on the ground observation vegetation phenology is 0.53 ** (*p* < 0.01), with a detected vegetation SOS of 1.26 days/year (*p* < 0.01). The vegetation SOS estimated by SOS_NDGI_ is the closest to the value retrieved from ground observation (Figure 6d).

### 3.3. Mechanism of Snow on Vegetation Green-Up

The survival analysis model system established SCF and SMD on vegetation SOS and quantitatively evaluated the contribution of SOS_NDVI_, SOS_EVI_, SOS_NDPI_, and SOS_NDGI_ of different environmental factors to vegetation growth mechanisms. The SOS_NDVI_ coefficient is −0.15, and the SOS_NDGI_ coefficient is −0.096. This indicates that the negative coefficient in the Cox model points towards a decrease in risk, and a negative coefficient implies that an increase in SOS_NDVI_ and SOS_NDGI_ of the vegetation SOS actually reduces the risk of mortality. The study used the standardized regression coefficient comparison method to analyze the impact intensity of various variables on the vegetation SOS, and the results showed that the vegetation SOS process exhibited differential response characteristics to various driving factors. It is worth noting that the dynamic effects of all variables on the green-up time exhibit significant time dependence, and their effect values change with time in accordance with the function relationship (ln (t)). The specific parameter characteristics are detailed in the quantitative analysis results in Table 2.

## 4. Discussion

### 4.1. Impact of Snow on Vegetation Green-Up

In this study, there was a strong correlation between the SOS derived from the four methods and ground observations; significant differences in the accuracy of the SOS obtained using various extraction methods were observed. Our results showed that the percentage of NDGI threshold for ground-observed SOS varied between 8% and 16%, depending on the vegetation type. This suggests that using a single relative threshold alone cannot ensure 100% accuracy in detecting SOS [36]. For instance, the percentages serving as thresholds in relation to CCR_max_ approximately 50% in the meadow steppe [37]. Therefore, CCR_max_ is more appropriate for detecting the beginning of vegetation SOS in its early stages, while CCR_max_ enhances vigorous photosynthesis and speeds up vegetation growth. In terms of the accuracy of extracting the SOS, CCR_max_ outperforms other methods. The research results are highly consistent with the research conclusions on winter wheat in the Huang Huai region and the grasslands of the Qinghai Tibet Plateau. This consistency not only validates the universality of research methods but also provides an important basis for the construction of cross-regional ecological models, indicating that there are common characteristics in the vegetation response mechanisms of different geographical units [38]. There are two potential factors contributing to CCR_max_’s superiority. Firstly, the vegetation indices threshold percentage obtained from the SOS by CCR_max_ closely matches that of ground observations. Secondly, CCR_max_ shows that it is less responsive to fluctuations in the initial value of the background vegetation indices. These initial values usually have a higher similar value affected by noise.

Previous research mainly focused on simulating factors such as snow depth, snow area, and snow melt date in vegetation SOS [39,40]. This study systematically explored the regulatory mechanism of SCF and SMD of vegetation phenology through empirical analysis that strictly excluded interference from other variables, revealing the key environmental factors that have not been fully studied in this field for a long time. This discovery not only supplements the study of snow response mechanisms but also provides a new model for mid-latitude ecosystems. This study reveals that in the environmental regulation system of the vegetation SOS in the snow-covered areas of the Mongolian Plateau, the seasonal snow depth exhibits a decisive regulatory role beyond traditional meteorological elements. This discovery is consistent with the theoretical framework of the coupling mechanism of snow water and heat effect—the process of SMD from late winter to early spring regulates the spatial and temporal heterogeneity of vegetation SOS by adjusting the surface energy. Shen et al. [41,42] studies have demonstrated that spring precipitation is a key driving variable for regional-scale vegetation SOS. Compared with the characteristics of rapid rainfall events, the water released by snow melting has a significant time lag effect [43,44]. Its progressive water supply mode forms a spatiotemporal coupling with the dynamic water demand of plant roots, especially during the SOS stage, which can effectively avoid water stress risks.

### 4.2. Analysis of the Mechanism of Snow Parameters on the Vegetation SOS

This study reveals that the response of vegetation SOS on the Mongolian Plateau to snow depth follows a nonlinear mechanism. The increase in snow depth before the season of the process of snow melting, forming surface energy balance, significantly delays the freeze-thaw cycle by inhibiting sensible heat exchange between soil and atmosphere [45,46]. This effect leads to a decrease in the rate of effective accumulated soil temperature, and at the same time, the physical cover layer formed by deep snow directly hinders the development of vegetation canopy. It is worth noting the snow cover albedo positive feedback mechanism. Delbart et al. [47] further strengthened this process, and the high albedo characteristics of snow cover reduce surface shortwave radiation absorption, resulting in a delayed soil warming rate compared to atmospheric warming during the same period.

This study elucidates that the snowmelt process, through its unique lag effect, constructs a sustained soil moisture supplement mechanism during the critical stage of vegetation growth, significantly promoting the dynamics of the evolution of the vegetation SOS period in alpine ecosystems. This effect is particularly prominent on the Mongolian Plateau, which is a typical ecosystem. The vegetation SOS process follows the snow water heat coupling model; the water flux released by the snow cover phase change not only compensates for the precipitation deficit in arid areas but also maintains the suitability of canopy temperature by regulating the surface evaporation effect. The underlying mechanism is that extending the snow cover period significantly improves vegetation water use efficiency by increasing the depth of meltwater infiltration and prolonging the effective water cycle [48,49,50].

### 4.3. Advantages and Limitations

This study innovatively introduces a survival analysis model and constructs a Cox hazards model to interpret the multi-scale driving mechanism of vegetation SOS on the Mongolian Plateau. This method breaks through the linear assumption constraints of traditional regression models and dynamically characterizes the nonlinear coupling effects of snow environmental factors through risk functions, demonstrating unique advantages in analyzing the spatiotemporal heterogeneity of SOS. The parameters quantify the response elasticity of the Mongolian Plateau ecosystem to extreme weather events. According to Diez et al. [51], the survival analysis model has significant superiority in simulating vegetation SOS. The nonlinear structure of this model can better capture the complexity of vegetation growth dynamics, thereby enhancing the sensitivity of the model to explanatory variables. In addition, survival analysis models can comprehensively consider multiple influencing factors and time factors and incorporate these variables into a unified framework for analysis. However, due to the complex terrain and the extremely uneven distribution of snow, the accuracy of these data are likely to be significantly diminished, thus restricting their utilization in comparable studies [52,53]. In contrast, the intercept of linear regression models is a fixed constant and cannot reflect spatial variations. The survival analysis model is applicable to datasets that have fluctuating and non-uniform observation frequencies. Moreover, it is especially well-suited for the analysis of substantial quantities of data that have been gathered from numerous locations and across various species [54]. However, due to the complex terrain and the extremely uneven distribution of snow, the accuracy of these data are likely to be significantly diminished, thus restricting their utilization in comparable studies [55,56,57,58]. In the future, investigations carried out in complex terrain scenarios might gain advantages from datasets with a higher resolution or from using alternative approaches. These would enable a more precise capture of the spatial changes in snow-related parameters. In addition, there is a key limitation, which is the use of multi-year average vegetation SOS to determine explanatory variables and simulate responses. Even though this approach streamlines the analysis procedure and achieves a balance between identifying representative SOS patterns and evading yearly fluctuations, making it easier to choose comparable explanatory variables, it is unable to comprehensively capture the interannual variations linked to large-scale climate changes. By implementing this approach, the robustness and sensitivity of phenological models with respect to specific situations can be improved, particularly when dealing with the scenario of constantly evolving climate conditions.

## 5. Conclusions

This research assessed the characteristics of the spatial distribution of the vegetation SOS and its temporal variation across the Mongolian Plateau. To verify the accuracy of satellite remote sensing SOS with observation phenology stations. Throughout the whole study region, SCF_Winter_ has a downward tendency. In terms of spatial distribution, between 2001 and 2023, there was a reduction of −0.2% in spatial SCF_Winter_. Furthermore, the spatial distribution of SCF_Winter_ shows a trend of decline from the northern part to the southern part.

Under climate warming, snow changes have had a significant impact on vegetation on the Mongolian Plateau. The fluctuations in snow parameters have the effect of not just modifying the climate near the surface but also exerting a profound influence on the ecological processes and events related to vegetation. After evaluating the accuracy of SOS, it was found that using different vegetation extraction methods would bring varying degrees of uncertainty in detecting SOS. The SOS of various vegetation types on the Mongolian Plateau generally takes place between DOY 58 and 195, which approximately coincides with the time from March to June. Among them, NDVI, EVI, NDPI, and NDGI methods can effectively capture SOS and maintain high accuracy in threshold percentage snow-covered areas. We also observed that the SOS derived from β_max_ exhibits the highest degree of agreement with ground-based observations.

## Figures and Tables

**Table 1:** Different steppe types on the Mongolian Plateau.

Number	Vegetation Type	Area (km^2^)
1	Meadow steppe	424,664.25
2	Typical steppe	1,640,985.75
3	Desert steppe	750,569.75

**Table 2:** Cox model coefficients for vegetation SOS on the Mongolian Plateau.

Influencing Variables	Coefficient	Standard Error	Z Statistic	*p*-Value
SCF	0.060	1.10	2.9	0.09
SCF × In(t)	0.008	1.00	2.8	0.09
SMD	0.020	1.00	1.9	0.17
SMD × In(t)	0.003	1.00	1.9	0.17
SOS_NDVI_	−0.150	0.86	7.6	<0.01
SOS_NDVI_ × In(t)	−0.020	0.98	7.7	<0.01
SOS_EVI_	−0.014	0.96	1.6	0.20
SOS_EVI_ × In(t)	−0.006	0.99	1.7	0.19
SOS_NDPI_	−0.020	0.98	0.62	0.43
SOS_NDPI_ × In(t)	−0.003	1.00	0.65	0.42
SOS_NDGI_	−0.096	0.91	8.2	<0.01
SOS_NDGI_ × In(t)	−0.013	0.99	8.3	<0.01

## Data Availability

Data available in a publicly accessible repository.

## Abbreviations

The following abbreviations are used in this manuscript:

SOS	Start of growing season
SCF	Snow cover fraction
SMD	Snow melt date
DEM	Digital elevation model
IGBP	International geosphere-biosphere programme
NDSI	Normalized difference snow index
NDVI	Normalized difference vegetation index
EVI	Enhanced vegetation index
NDPI	Normalized difference phenology index
NDGI	Normalized difference greenness index
CREN	Chinese ecosystem research network
RMSE	Root mean square error
DOY	Day of the year
